# Design of readers for pathological PTM proteoforms in Alzheimer’s disease

**DOI:** 10.64898/2026.10.20.760520

**Published:** 2026-10-21

**Authors:** Kejia Wu, Tiana Schantz, Lemuel Chang, Prarthna Chabria, Elaina Bashaw, Thejesh Reddy Kalatturu, Doo Yeon Kim, Anqi Chen, Nathaniel Corley, Asim K. Bera, Alex Kang, Hannah Nguyen, Johnny Mendoza, Xinting Li, Magnus S. Bauer, Misa Baum, Brian C. Kraemer, Yuyu Song, David Baker

## Abstract

Alzheimer’s disease (AD) is characterized by heterogeneous Tau phosphorylation and aberrant amyloid-β (Aβ) processing, yet molecular tools that distinguish closely related molecular states of tau and Aβ with predefined specificity remain difficult to develop. Here, we designed de novo protein readers targeting three disease-associated phospho-Tau regions and N-terminal pyroglutamate-modified Aβ (N3pG-Aβ). Screening 192 designs per target identified 51 modification-selective hits among 768 candidates (6.6%). Representative readers exhibited nanomolar to subnanomolar affinities for modified peptides, discriminated authentic modifications from unmodified or substitution-based controls, and preferentially recognized modified full-length targets. We further programmed local modification specificity by interface redesign, generating pT231 readers conditional on the phosphorylation state of neighboring S235 and S237. A 2.0 Å crystal structure of an N3pG-Aβ reader closely matched the computational design model (interface Cα RMSD = 0.52 Å), and redesign of pT231 readers tuned apparent binding affinities from greater than 1 μM to below 1 nM. In a human 3D neuronal culture with both pathological Aβ and tau, selective readers targeting N3pG-Aβ, Tau pT231, and Tau pT217 recognized disease-associated signals, exhibiting colocalization with related antibodies and much higher signal in the AD line than the control. Tau pT231 readers further quantitatively distinguish AD patients and control human brain lysates in dot-blot assays. Together, these results establish genetically encoded readers with programmable specificity for defined Tau and Aβ modification states, enabling molecular-state-resolved detection in human disease models and biological samples, and lay a foundation for controlled pathological molecular perturbation.

